# Real‐World Experience With Control‐IQ Technology in Saudi Children With Insulin‐Dependent Diabetes: A Single‐Center Observational Study

**DOI:** 10.1155/pedi/6113065

**Published:** 2026-03-01

**Authors:** Afaf Alsagheir, Bassam Bin-Abbas, Razan Alsagheir, Raghad Alhuthil, Aseel Aljuwair, Norah H. Alobailly, Sheikhah Almoaily

**Affiliations:** ^1^ Department of Pediatrics, King Faisal Specialist Hospital and Research Centre, Riyadh, Saudi Arabia, kfshrc.edu.sa; ^2^ College of Medicine, Alfaisal University, Riyadh, Saudi Arabia, alfaisal.edu; ^3^ Department of Nursing Development and Saudization, King Faisal Specialist Hospital and Research Centre, Riyadh, Saudi Arabia, kfshrc.edu.sa; ^4^ Department of Diabetes Education, VitalAire Arabia Company, Riyadh, Saudi Arabia

**Keywords:** glycemic control, insulin pump, insulin-dependent diabetes, pediatrics, Saudi Arabia

## Abstract

**Objectives:**

The Tandem t:slim X2 insulin pump with the Control‐IQ technology (Control‐IQ) is an automated insulin delivery (AID) system for glycemic control but has limited data in Saudi pediatric and young adult populations. We aim to evaluate the efficacy and safety of Control‐IQ therapy in children with insulin‐dependent diabetes and previously treated with multiple daily injections (MDIs). The primary outcome is to assess the change in HbA1c 6 months after initiating Control‐IQ technology.

**Methods:**

This prospective observational study evaluated children aged 2–14 years with insulin‐dependent diabetes who transitioned from treatment with MDI to the Control‐IQ technology in the diabetes clinic at the King Faisal Specialist Hospital and Research Centre (KFSHRC) in Saudi Arabia.

**Results:**

A total of 100 patients (44 boys and 56 girls; median age was 11 years) were included. Most (82%) had a history of severe hypoglycemia. Following Control‐IQ initiation, median HbA1c significantly decreased from 9.2% to 6.9% at 6 months (−25.0%; *p* < 0.001), accompanied by a 24.4% reduction in the daily insulin dose. Time in range (TIR) (70–180 mg/dL) improved from 45.6% to 68.8% (+23.2%; *p* < 0.001), while time in significant hypoglycemia (<54 mg/dL) decreased by 88.9% (*p* < 0.001). At 6 months, 83% achieved ≥60% TIR, and 54% reached ≥70%. Adherence and engagement were high, with no severe hypoglycemia or discontinuations. Only 3% experienced mild/moderate ketoacidosis due to technical issues in the Control‐IQ.

**Conclusions:**

The findings demonstrated that Control‐IQ technology is both effective and safe in improving glycemic outcomes in children with insulin‐dependent diabetes in a real‐world clinical setting. However, successful implementation requires comprehensive training and continuous support.

## 1. Introduction

Technological advancements such as continuous glucose monitoring (CGM), insulin pumps, and hybrid closed‐loop (HCL) automated insulin delivery (AID) systems, particularly the Tandem t:slim X2 insulin pump with Control‐IQ technology (Control‐IQ), have significantly improved the management of type 1 diabetes (T1D), especially by enhancing glycemic control and reducing hypoglycemia [1, 2]. While randomized controlled trials (RCTs) have demonstrated the efficacy of these systems, real‐world outcomes, particularly in pediatric populations, often fall short due to variability in user behavior, device usability, and healthcare infrastructure [3, 4].

In children, the success of insulin pump therapy is influenced by multiple factors, including device complexity, sensor accuracy, ease of use, and caregiver involvement. Barriers such as cost, limited access to structured education, provider hesitancy, and caregiver burden may hinder optimal use and outcomes [1, 3]. These challenges are often amplified in real‐world settings, where long‐term follow‐up and consistent support systems are frequently lacking—particularly in socioeconomically underrepresented or resource‐limited populations [3, 5]. Additionally, the transition from multiple daily injections (MDIs) to hybrid AID systems can present notable barriers. Families often report technological frustrations, alarm fatigue, and a lack of trust in automation, especially when caregivers are responsible for device management in younger children [5].

While studies from Western populations support the clinical benefits of Control‐IQ technology [6], there remains a significant gap in evidence from underrepresented regions, including Saudi Arabia, where T1D incidence is rising [7]. To date, only two local studies have evaluated insulin pump outcomes in children, both demonstrating HbA1c improvements with continuous subcutaneous insulin infusion (CSII) over MDI [8, 9]; however, neither study investigated AID systems.

Moreover, regulatory bodies like the European Medicines Agency and the US Food and Drug Administration (FDA) now place growing value on real‐world studies as a way to complement RCTs [10–12]. Thus, real‐world data provide insight into how technologies perform in everyday clinical settings over the long term, helping to guide regulatory decisions and support the broader use of technological advancements in managing pediatric diabetes [12].

This study aims to evaluate the efficacy and safety of the Control‐IQ technology in Saudi children with insulin‐dependent diabetes transitioning from MDI. By assessing real‐world outcomes, particularly changes in HbA1c at 6 months post the Control‐IQ technology, this research seeks to generate clinically meaningful data that can inform diabetes management strategies in the region.

## 2. Materials and Methods

This prospective observational study investigated the transition of patients with insulin‐dependent diabetes from MDI to the Control‐IQ technology as part of their routine management. The study was conducted at the Pediatric Endocrinology Clinic of King Faisal Specialist Hospital and Research Centre (KFSHRC), a tertiary care center. On average, the clinic manages ~500 pediatric patients with T1D annually.

Verbal consent and minor assent were obtained from all participants and their guardians in the study. Ethical approval was granted by the ethics committee at KFSHRC (Reference: 2231203).

### 2.1. Study Population

**Figure 1:**
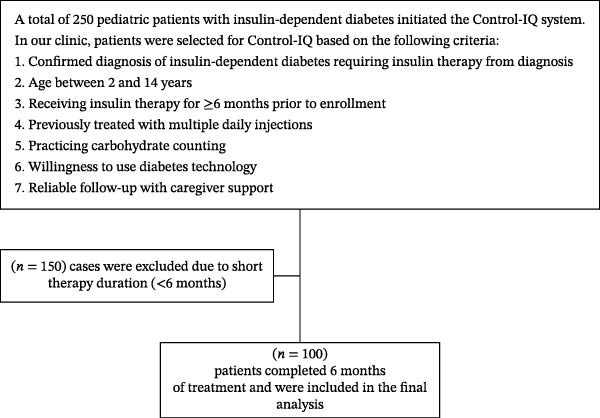
Flowchart of subjects’ enrollment.

Participants were eligible for enrollment if they were children aged 2–14 years with a confirmed diagnosis of insulin‐dependent diabetes mellitus requiring insulin therapy since diagnosis and had been receiving insulin treatment for at least 6 months prior to enrollment. All participants were previously managed using MDI, were practicing carbohydrate counting, and demonstrated willingness to adopt diabetes technology, with reliable follow‐up ensured through caregiver support. Participants were excluded if they had type 2 diabetes or post‐transplant diabetes, had previously used Control‐IQ technology, or had less than 6 months of follow‐up after device initiation (Figure 1). None of the enrolled participants had prior experience with insulin pump therapy in open‐loop mode; however, most had intermittently used CGM before enrollment.

### 2.2. Training and Monitoring

Our participants used the Dexcom G6 CGM system, which was initiated at least 2 weeks prior to starting the Control‐IQ technology to allow for CGM‐only data collection and patient acclimatization. During this CGM‐only period, baseline glucose patterns were assessed to support individualized pump settings.

Training and monitoring were delivered through individualized sessions tailored to each family’s needs and learning pace. The program included three sessions: an introductory session on pump and CGM technology (1–1.5 h), a hands‐on training on pump operation, settings, and safety features (2 h), and a follow‐up session focused on review and troubleshooting (1 h), totaling ~4–4.5 h over 3 days. Training was conducted in person by a certified diabetes educator with expertise in Control‐IQ technology and included both the child and at least one parent or caregiver. To support ongoing learning, families were also provided with educational videos and printed booklets for home reference.

Following training, patients were transitioned to the Control‐IQ and subsequently monitored remotely every 1–2 weeks for the first 1–2 months and then monthly thereafter. The Control‐IQ settings were adjusted based on real‐time CGM data obtained during the study period. Furthermore, participants attended clinic follow‐up visits every 3 months, during which serum HbA1c levels were measured for ongoing monitoring.

### 2.3. Control‐IQ Algorithm Setup and Adjustment Protocol

At initiation, the Control‐IQ technology was activated for all patients, with pump settings (basal rates, insulin‐to‐carbohydrate ratios, and correction factors) individualized based on prior insulin regimens and CGM data. During the first 2–4 weeks, algorithm parameters were closely monitored and fine‐tuned through weekly follow‐up calls, remote CGM data reviews (uploaded by patients’ families), and an in‐clinic visit at 1 month for formal evaluation and necessary adjustments. In the stable phase following the first month, algorithm settings were typically reviewed and modified every 1–3 months, or sooner if the patient reported recurrent hypoglycemia or hyperglycemia, or if CGM pattern analysis suggested the need for further optimization.

### 2.4. Data Collection

Data were systematically collected between May 2023 and July 2024 by pediatric endocrinologists, using the Diasend system (Glooko Inc., Mountain View, CA, USA), which integrates data from most CGM devices and Tandem’s Control‐IQ system. Additional clinical and laboratory data were retrieved from the KFSHRC electronic health information system. For each participant, baseline data included demographic information (age and sex), diabetes duration, previous insulin regimen, total daily insulin dose, and baseline HbA1c. Glycemic outcomes were assessed using CGM metrics, with baseline values taken from the 2 weeks prior to initiating Control‐IQ and postintervention data averaged over the last 3 months of device use. Primary glycemic outcomes included time in range (TIR), defined as the percentage of time sensor glucose levels remained between 70 and180 mg/dL. Additional metrics included the percentage of time in hypoglycemia (<70 mg/dL) and the coefficient of variation (%CV). Safety outcomes included the frequency of severe hypoglycemia (requiring assistance due to seizures or loss of consciousness) and documented episodes of diabetic ketoacidosis (DKA). Compliance with follow‐up was defined as attending ≥80% of scheduled clinic or virtual visits and maintaining active CGM data uploads throughout the 6‐month period.

### 2.5. Statistical Analysis

Our study included all eligible patients who completed a 6‐month follow‐up (*n* = 100). A post hoc power analysis was performed based on a paired *t*‐test using the observed standard deviation (SD) of the within‐subject HbA1c differences (1.6%). With 100 paired observations and a two‐sided *α* of 0.05, the study had 87.2% power to detect a 0.5% absolute reduction in HbA1c, 99.1% power to detect a 0.7% reduction, and >99.9% power to detect a 1.0% reduction.

Data were analyzed using STATA software (StataCorp LLC, College Station, TX, USA) Version 18. Categorical data were expressed as frequencies and percentages, while continuous data were expressed as median and interquartile range (IQR) or mean and SD, depending on data distribution. Normality was assessed using the Kolmogorov–Smirnov test. Statistical significance was determined using the Wilcoxon signed‐rank test or paired *t*‐test as appropriate. No adjustment for multiple comparisons was planned or performed.

## 3. Results

Of 250 cases on Control‐IQ technology, 100 cases met the inclusion criteria and were included in this study (Figure 1). They had a median age of 11 [9−13] years and consisted of 44 boys (44.0%) and 56 girls (56.0%). The youngest enrolled patient was 2 years old. Severe recurrent hypoglycemia at baseline was observed in 82 individuals (82.0%). In terms of DKA episodes prior to treatment, 73% had no prior DKA, while 27% experienced between one and three episodes (Table 1).

**Table 1:** Background information of study participants (*n* = 100).

Characteristics	*n* (%)
Sex	
Male	44 (44.0)
Female	56 (56.0)
Age at diagnosis (years), mean ± SD	6.4 ± 2.7
Current age (years), median [IQR]	11 [9−13]
Duration from diagnosis to Control‐IQ start (years), median [IQR]	4 [2−5]
Aged ≤5 years at start of insulin injections	38 (38.0)
Aged ≤5 years at start of Control‐IQ	7 (7.0)
Previous Control‐IQ experience	0 (0.0)
Positive family history	23 (23.0)
Previous medications	
Tresiba and aspart	100 (100.0)
Comorbidities	
None	65 (65.0)
Congenital heart disease	4 (4.0)
End‐stage renal disease	3 (3.0)
Obesity	4 (4.0)
Nephrotic syndrome	2 (2.0)
Wolfram syndrome	2 (2.0)
Celiac disease	3 (3.0)
Postpancreatectomy	2 (2.0)
Development delay	2 (2.0)
Other^a^	13 (13.0)
Severe hypoglycemia before Control‐IQ^b^	82 (82.0)
Diabetic ketoacidosis episodes before Control‐IQ	
0	73 (73.0)
1	13 (13.0)
2	10 (10.0)
3	4 (4.0)

Abbreviations: IQR, interquartile range; SD, standard deviation.

^a^This includes patients with a history of stem cell or solid organ transplantation, leukemia, systemic lupus erythematosus (SLE), autoimmune polyendocrine syndrome type 1 (APS‐1), Graves’ disease, myositis, hypothyroidism, or seizure disorders.

^b^Severe event that requires assistance due to loss of consciousness or seizures.

**Figure 2:**
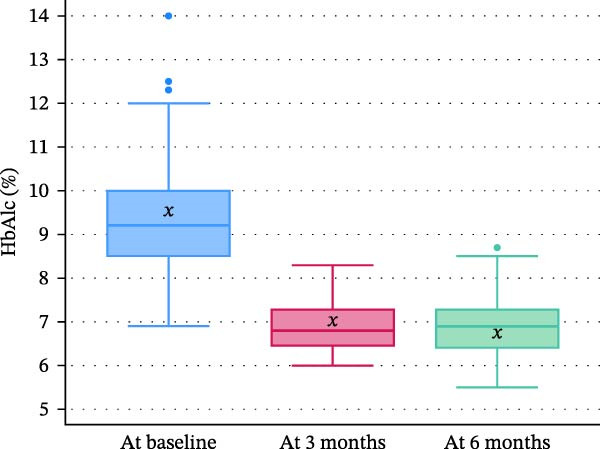
Change in HbA1c (%) following Control‐IQ Initiation. Box plots display the distribution of HbA1c levels at baseline, 3 months, and 6 months after initiating Control‐IQ. For each time point, the central horizontal line represents the median, the box bounds indicate the first (Q1) and third quartiles (Q3), and the whiskers extend to the minimum and maximum values within 1.5× the interquartile range (IQR). Outliers are shown as individual points, and the “*x*” symbol denotes the mean.

Significant improvements were observed in glycemic control following the Control‐IQ technology. The median HbA1c significantly decreased from 9.2% [IQR: 8.5–10.0] at baseline to 6.8% [IQR: 6.45–7.3] at 3 months (−2.4 difference [−26.1%]; *p* < 0.001) and to 6.9% [IQR: 6.4–7.3] at 6 months (−2.3 difference [−25.0%]; *p* < 0.001). Corresponding mean values were 9.5 ± 1.6, 6.9 ± 0.6, and 6.9 ± 0.7, respectively, indicating sustained improvement in glycemic control (Figure 2). In parallel, the total daily insulin dose decreased by 24.4%, from 0.90 [0.80–1.15] to 0.68 [0.54–0.87] units/kg (*p* < 0.001) (Table 2).

**Table 2:** Glycemic and clinical outcomes following Control‐IQ initiation (*n* = 100).

Outcome	Baseline	3 months	6 months	*p*‐Value^a^
HbA1c (%), median [IQR]	9.2 [8.5–10.0]	6.8 [6.45–7.3]	6.9 [6.4–7.3]	<0.001
Weight (kg)	39.0 [29.7–48.1]	—	40.0 [29.6–49.0]	0.012
BMI	18.1 [16.7–20.6]	—	18.4 [16.5–21.0]	0.514
Total daily insulin dose (units/kg), median [IQR]	0.90 [0.80–1.15]	—	0.68 [0.54–0.87]	<0.001
Time in range 70–180 mg/dL (%), mean ± SD	45.6 ± 11.9	—	68.8 ± 10.4	<0.001
Time below range <70 mg/dL (%), median [IQR]	4 [3–4]	—	2 [1–3]	<0.001
Time below range <54 mg/dL (%), mean ± SD	3.24 ± 2.09	—	0.36 ± 0.60	<0.001
Time above range >180 mg/dL (%), mean ± SD	—	20.4 ± 5.8	19.2 ± 4.8	—
Time above range >250 mg/dL (%), median [IQR]	—	9 [6–13]	8 [4–13]	—
Achieved time in range ≥60% of the time, *n* (%)	—	81 (81.0)	83 (83.0)	—
Achieved time in range ≥70% of the time, *n* (%)	—	42 (42.0)	54 (54.0)	—
Duration on Control‐IQ (months), median [IQR]	—	—	7 [6–8]	—
Coefficient of variation (%), mean ± SD	—	37.8 ± 7.1	36.8 ± 6.1	—
Adherent to data downloads^b^, *n* (%)	—	—	82 (82.0)	—
Responsive to follow‐up calls, *n* (%)	—	—	87 (87.0)	—
Following clinical recommendations, *n* (%)	—	—	78 (78.0)	—
Severe hypoglycemia on Control‐IQ, *n* (%)	—	—	0 (0.0)	—
Adverse symptoms following insulin pump, *n* (%)	—	—	0 (0.0)	—
Diabetic ketoacidosis on Control‐IQ, *n* (%)	—	—	3 (3.0)	—
Discontinued Control‐IQ, *n* (%)	—	—	0 (0.0)	—

*Note:* HbA1c, glycated hemoglobin.

Abbreviations: IQR, interquartile range; SD, standard deviation.

^a^ *p*‐Value compares baseline to 6 months using the Wilcoxon signed‐rank test or paired *t*‐test as appropriate.

^b^Any patient’s family who uploads their reports 80% of the time requested during the visit is considered adherent.

At 3 months postinitiation, the mean percentage of time in the high glucose range was 20.4% ± 5.8%, which improved slightly to 19.2% ± 4.8% at 6 months. The median time in very high glucose levels (>250 mg/dL) declined from 9% [6–13] to 8% [4–13]. Additionally, time spent in hypoglycemia (<70 mg/dL) was reduced from 4% [3,4] to 2% [1–3] (Table 2).

Further improvements were noted in overall glycemic metrics. TIR (70–180 mg/dL) increased by 23.2% points, from 45.6% ± 11.9% to 68.8% ± 10.4% (*p* < 0.001), while time in significant hypoglycemia (<54 mg/dL) decreased by 88.9%, from 3.24% ± 2.09% to 0.36% ± 0.60% (*p* < 0.001). In terms of anthropometric outcomes, a modest but statistically significant increase in weight was observed (+1.0 kg, *p* = 0.012). However, the change in BMI was minimal and not statistically significant (+0.25, *p* = 0.514) (Table 2).

Most participants achieved target glycemic metrics: 81% reached ≥60% TIR at 3 months, increasing to 83% at 6 months. Likewise, the proportion achieving ≥70% TIR rose from 42% to 54%. The median duration of Control‐IQ use was 7 months [IQR: 6–8] (Table 2).

Glycemic variability, as measured by the CV, slightly improved from 37.8% ± 7.1% at 3 months to 36.8% ± 6.1% at 6 months. Adherence and engagement were generally high, with 82% consistently uploading data, 87% responsive to follow‐up calls, and 78% following clinical recommendations. Importantly, no severe hypoglycemia or adverse events were reported. Only three participants (3%) experienced mild‐to‐moderate DKA related to technical issues, and none discontinued Control‐IQ during the follow‐up period (Table 2).

## 4. Discussion

This study evaluated the efficacy and safety of the Control‐IQ technology in children with insulin‐dependent diabetes previously treated with MDI. Our primary outcome is glycemic control at 6 months as measured by HbA1c, which showed a clinically and statistically significant improvement both at 3 months and at 6 months, representing a 26.1% and 25% reduction, respectively. This improvement is notably greater than the 0.5%–0.6% reduction typically reported in RCTs of Control‐IQ technology [13, 14], possibly due to the higher baseline HbA1c and transition from MDI to advanced insulin delivery systems in our cohort.

This enhancement in HbA1c was accompanied by a reduction in total daily insulin dose (−24.4%), suggesting improved insulin efficiency and likely reflecting better algorithm‐guided dosing. Additionally, TIR (70–180 mg/dL) improved by 23% points, from 45.6% to 68.8%, while glycemic variability, as measured by %CV, also declined. These findings support the growing body of evidence demonstrating that Control‐IQ enhances both average glucose and glucose stability [15, 16].

Importantly, safety outcomes were favorable. Time spent in significant hypoglycemia (<54 mg/dL) dropped by nearly 89%, and no episodes of severe hypoglycemia were reported during the follow‐up period. Although three participants experienced mild DKA events due to technical issues, no one discontinued Control‐IQ, suggesting overall acceptability and safety in this population. These results are consistent with prior studies that report reduced rates of acute complications and hypoglycemia burden using Control‐IQ [17–19].

Beyond the numbers, the patient and caregiver experience plays a crucial role in the success of diabetes technologies. In our real‐world cohort, adherence to device use and follow‐up was high: 82% uploaded data consistently, 87% responded to follow‐up, and 78% followed clinical recommendations. These findings likely reflect the structured education and support provided. However, transitioning to HCL systems can be challenging, especially for younger children, due to alarm fatigue, technical troubleshooting, and the need for consistent caregiver involvement. Several studies have highlighted that although Control‐IQ can reduce diabetes‐related stress and improve sleep quality, it may initially increase caregiver burden before confidence in automation builds [20, 21].

Our results also align with international experiences. A recent UK real‐world study reported significant reductions in HbA1c and hypoglycemia frequency after 12 months of Control‐IQ use, alongside improvements in family quality of life [16]. Similarly, studies in Belgium have shown comparable positive outcomes [12, 15]. A multicenter pediatric study by Meulemeester et al. [15] found that Control‐IQ improved TIR from 51.6% to 64.4% and HbA1c from 7.8% to 7.1%. This study also reported enhancements in quality of life and a reduction in school and work absences [12]. Additionally, another Belgian study on adult users of Control‐IQ demonstrated a reduction in time spent <70 mg/dL from 4.2% to 1.9% over 1 year, which aligns with the hypoglycemia data observed in this pediatric cohort [15].

Additional studies support these benefits. In children aged 2–6 years, Control‐IQ increased TIR from 56% to 68% without raising hypoglycemia risk [22]. A meta‐analysis of children and adults studies found that the technology leads to average gains of 2.8 h/day in TIR and reductions in HbA1c and mean glucose levels. They also highlighted that individuals with poor baseline glycemic control tend to benefit most, underscoring the importance of personalized treatment selection [6]. Families also reported improved sleep, reduced parenting stress, and fewer hypoglycemia worries [23]. Real‐world users consistently show higher satisfaction and quality of life [24], and a Swedish cost‐effectiveness study found Control‐IQ to be cost‐saving compared to MDI across long‐term projections [25].

## 5. Limitations

The single‐center of this study limited its generalizability. Future studies should be conducted on a larger scale and with longer follow‐up to derive nationwide conclusions and understand the long‐term impact of Control‐IQ technology on children with insulin‐dependent diabetes and their caregivers. Finally, no adjustment for multiple comparisons was planned or performed; therefore, *p*‐values for secondary outcomes should be interpreted with caution. Furthermore, future studies should evaluate the patient experience and caregiver perspectives to obtain valuable insights for optimizing treatment approaches and improving patient care.

## 6. Conclusion

In conclusion, our findings demonstrate that Control‐IQ technology is both effective and safe in improving glycemic outcomes in children with insulin‐dependent diabetes in a real‐world clinical setting. These improvements were achieved without increasing the risk of hypoglycemia or DKA and were supported by high levels of patient and caregiver engagement. However, successful implementation requires comprehensive training and continuous support.

## Author Contributions

**Afaf Alsagheir and Bassam Bin-Abbas:** conceptualization, formal analysis, writing – original draft. **Razan Alsagheir and Aseel Aljuwair:** data curation, methodology, writing – original draft. **Sheikhah Almoaily:** Investigation, methodology, writing – original draft. **Raghad Alhuthil:** formal analysis, methodology, writing – review and editing. **Norah H. Alobailly:** data curation, investigation, writing – review and editing.

## Funding

The authors have nothing to report.

## Disclosure

Patients and/or the public were involved in the design, or conduct, or reporting, or dissemination plans of this research. Refer to Section 2 for further details. All authors reviewed and approved the final draft. An earlier version of this study was presented as an e‐poster at (17th International Conference on Advanced Technologies & Treatments for Diabetes, 06.03.2024), and it can be accessed via the following link: https://cslide.ctimeetingtech.com/attd24/attendee/eposter/poster/1322.

## Conflicts of Interest

The authors declare no conflicts of interest.

## Data Availability

The data that support the findings of this study are available upon request from the corresponding author. The data are not publicly available due to privacy or ethical restrictions.
